# Redefining borders in the sensorimotor cortex

**DOI:** 10.7554/eLife.110364

**Published:** 2026-02-09

**Authors:** Sylvain Crochet

**Affiliations:** 1 Laboratory of Sensory Processing, Brain Mind Institute, School of Life Sciences, Ecole Polytechnique Fédérale de Lausanne (EPFL) Lausanne Switzerland

**Keywords:** brain mapping, sensorimotor cortex, motor control, Mouse

## Abstract

Experiments mapping individual neurons in the sensorimotor cortex of mice show that sharp transitions in functional properties can define cortical regions.

**Related research article** Salimian S, Grier HA, Kaufman MT. 2025. Neural activity profiles reveal overlapping, intermingled subpopulations spanning area borders in mouse sensorimotor cortex. *eLife* **14**:RP109240. doi: 10.7554/eLife.109240.

The human cortex is divided into anatomically and functionally distinct regions, such as the visual cortex for sight and the motor cortex for movement. This division has long been a topic of debate in neuroscience. It assumes that each anatomically defined area corresponds to a specific function. However, the exact boundary between these regions is often unclear. While anatomical criteria can help outline the different parts of the cortex to some degree, defining them functionally is much more difficult, especially for frontal motor and premotor regions. This calls for a reevaluation of how these regions are defined and understood.

Sensory and motor areas are traditionally mapped using sensory-evoked activity and local stimulation, respectively. However, the classical segregation between sensory and motor cortices has been increasingly challenged: sensory-evoked responses are not confined to sensory areas, motor-related activity preceding the initiation of movement has been reported across much of the cortex (Oryshchuk et al., 2024; Stringer et al., 2018), and movements can be evoked by stimulation of both sensory and motor regions (Matyas et al., 2010; Penfield and Boldrey, 1937; Tamura et al., 2025). As a result, the functional organization of the cortex – and the delimitation of its different regions – has become increasingly blurred.

This ambiguity is especially apparent in the frontal cortex of mice. Even widely used resources such as the Allen Mouse Brain Atlas divide the dorsal frontal cortex into primary and secondary motor regions in a rather coarse manner, despite ample evidence supporting a finer-grained organisation. For example, both the anterolateral motor cortex, which is part of the secondary motor cortex, and the tongue and jaw primary motor cortex are involved in the process of licking. Although activity increases in both regions before licking begins, the tongue and jaw primary motor cortex consistently encodes the kinematics of individual licks, whereas the anterolateral motor cortex appears to encode higher-level variables related to the motor plans (Guo et al., 2014; Oryshchuk et al., 2024; Xu et al., 2022). These findings provide strong evidence for a functional specialization within the frontal cortical areas and raise the question of how we can identify meaningful functional boundaries in such distributed and overlapping neural systems.

Now, in eLife, Sohrab Salimian, Harrison Grier and Matthew Kaufman from the University of Chicago report the results of experiments on mice that could help us answer this question (Salimian et al., 2025). Salimian et al. surveyed neuronal activity across a substantial portion of the sensorimotor cortex, recording the activity of single neurons in head-fixed mice performing a multi-target reaching task. In total, responses from more than 39,000 neurons were recorded, enabling the researchers to develop quantitative metrics for fine-scale functional mapping of the imaged cortical regions (Figure 1).

**Figure 1.**
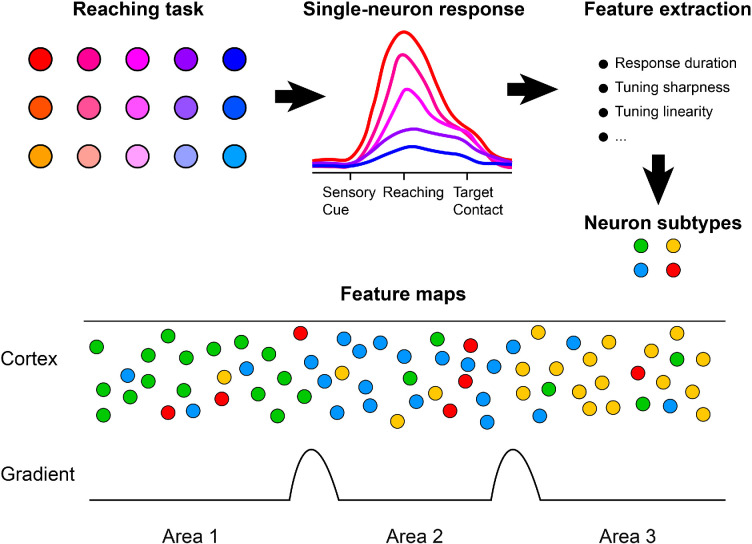
Functional delineation of different areas in the sensorimotor cortex. Salimian et al. imaged the activity of individual neurons in the sensorimotor cortex of mice while the animals performed a task that involved reaching for different target positions. This schematic shows how the responses of individual neurons (top left and middle) were used to extract functional features (such as response duration and tuning sharpness; top right), which in turn were used to define different neuron subtypes (green, yellow, red and blue circles). Mapping the changes in different functional features revealed spatial gradients (bottom) with sharp transitions between different areas. Moreover, although the different subtypes tend to be found in specific areas, each area also contains several different subtypes.

The results confirm a growing body of work showing that, at the single-neuron level, diverse response types are found in all cortical areas, albeit in different proportions. As expected, fine-grained aspects of movement, such as target location, were more strongly represented in motor areas than in sensory regions, and neurons in the frontal secondary motor cortex exhibited more complex and multimodal response profiles.

Crucially, Salimian et al. went beyond classifying neurons by broad task-related activity, such as movement, sensory, decision or reward signals. Instead, they extracted the response characteristics of individual neurons (such as response duration and tuning sharpness) and classified them into different neuronal subtypes (Figure 1). Mapping the response characteristics across the dorsal frontal cortex revealed spatial gradients with sharp transitions between different cortical regions.

These transitions defined clear functional boundaries. Some matched known anatomical borders, such as those surrounding a region known as forepaw primary somatosensory cortex (which receives sensory input from the forepaw) and the borders that separate the primary somatosensory cortex, the primary motor cortex and the secondary motor cortex. However, other transitions exposed previously unappreciated subdivisions, including distinct subregions within primary somatosensory and motor cortices. Thus, rather than assuming predefined borders, the study demonstrates that functional boundaries can emerge from gradual, spatially organized changes in neuronal population properties.

Importantly, sharp functional boundaries do not mean that individual cortical areas are composed of uniform or unique neuronal populations. On the contrary, Salimian et al. show that functionally defined neuronal subtypes are widely distributed and intermingled across area borders (Figure 1), supporting the view that cortical specialization arises from graded changes in neural population structure rather than strict segregation of regions.

Together, this work provides compelling evidence that distinct functional boundaries between cortical regions can be identified using population-level neuronal features, even when individual neuron responses overlap. It also opens promising avenues for refining functional maps of the mouse sensorimotor cortex when using a broader range of sensory inputs and behaviors. An open question is whether such functionally defined boundaries can be incorporated into a widely adopted reference atlas of the cortex.
